# Transcriptome Profiling of Human Ulcerative Colitis Mucosa Reveals Altered Expression of Pathways Enriched in Genetic Susceptibility Loci

**DOI:** 10.1371/journal.pone.0096153

**Published:** 2014-05-01

**Authors:** Christopher J. Cardinale, Zhi Wei, Jin Li, Junfei Zhu, Mengnan Gu, Robert N. Baldassano, Struan F. A. Grant, Hakon Hakonarson

**Affiliations:** 1 Center for Applied Genomics, Perelman School of Medicine at the University of Pennsylvania, Philadelphia, Pennsylvania, United States of America; 2 Department of Computer Science, New Jersey Institute of Technology, Newark, New Jersey, United States of America; 3 Division of Gastroenterology, Hepatology, and Nutrition, Children’s Hospital of Philadelphia, Perelman School of Medicine at the University of Pennsylvania, Philadelphia, Pennsylvania, United States of America; 4 Department of Pediatrics, Perelman School of Medicine at the University of Pennsylvania, Philadelphia, Pennsylvania, United States of America; University College Dublin, Ireland

## Abstract

Human colonic mucosa altered by inflammation due to ulcerative colitis (UC) displays a drastically altered pattern of gene expression compared with healthy tissue. We aimed to understand the underlying molecular pathways influencing these differences by analyzing three publically-available, independently-generated microarray datasets of gene expression from endoscopic biopsies of the colon. Gene set enrichment analysis (GSEA) revealed that all three datasets share 87 gene sets upregulated in UC lesions and 8 gene sets downregulated (false discovery rate <0.05). The upregulated pathways were dominated by gene sets involved in immune function and signaling, as well as the control of mitosis. We applied pathway analysis to genotype data derived from genome-wide association studies (GWAS) of UC, consisting of 5,584 cases and 11,587 controls assembled from eight European-ancestry cohorts. The upregulated pathways derived from the gene expression data showed a highly significant overlap with pathways derived from the genotype data (33 of 56 gene sets, hypergeometric *P* = 1.49×10^–19^). This study supports the hypothesis that heritable variation in gene expression as measured by GWAS signals can influence key pathways in the development of disease, and that comparison of genetic susceptibility loci with gene expression signatures can differentiate key drivers of inflammation from secondary effects on gene expression of the inflammatory process.

## Introduction

The inflammatory bowel diseases (IBD)–Crohn’s disease (CD) and ulcerative colitis (UC)–are chronic disorders resulting in autoimmune destruction of segments of the gastrointestinal tract. The study of these disorders has benefited from recent technological advances that enable transcriptome quantification on microarrays, including distinguishing inflammatory expression patterns in healthy tissue from tissue derived from various levels of disease activity. One goal of gene expression profiling has been to identify dysregulated proteins which are rooted in the pathogenesis of the disease and may serve as targets for therapeutic intervention. A second goal has been to classify samples in order to support a particular diagnosis when the IBD subtype is ambiguous since each will require distinct medical and surgical treatment. These studies have generally shown that ulcerative colitis mucosa has a pattern of gene expression that is distinguishable from healthy tissue, while colonic mucosa from Crohn’s disease or from tissue not macroscopically involved in the disease process can have patterns of gene expression that are inflammatory, normal, or a degree of mixture of both [1]–[4].

Parallel with these efforts to characterize the UC transcriptome, microarray genotyping technology has been applied to genome-wide association studies (GWAS) in order to determine which single nucleotide polymorphisms (SNP) confer a hereditary predisposition to developing IBD. The most recent meta-analysis of studies performed to date identified 163 loci as either risk-conferring or protective in CD, UC, or both [5]. Linkage studies highlighted the role of the HLA region and *NOD2* [6], [7], and later GWAS implicated *IL23R* [8], *ATG16L1* [9], *IRGM*, *NKX2–3*, *TNFSF15* [10] among the lead genes involved in the disease process together with many others [11], [12]. The implicated loci clearly illustrate the importance of cytokine biology, such as the IL12/23 pathway [13] and the tumor necrosis factor superfamily. Other functions of interest are epithelial barrier function, autophagy, and interactions with the gut microbiome [14], [15].

In this study we integrated both gene expression profiling in colonoscopy biopsies of UC with genome wide genotype analyses in order to identify and prioritize the gene sets and molecular pathways most consistently associated with UC. Our results showed that a large number of gene sets are differentially regulated between UC and normal biopsies. We conducted a genome-wide association study (GWAS) in order to identify gene sets using a hypergeometric test and found that most of the pathways identified were concordant with the transcriptome data. Comparison of transcriptome and GWAS data can allow us to delineate initiators of the disease process from secondary markers of the inflammatory process observable on gene expression arrays. The functional significance of the pathways and their implication for understanding IBD pathogenesis will be an important future research area in IBD.

## Results

### Gene Expression Data Set Description

We obtained three published data sets from the Gene Expression Omnibus (GEO) of the NIH, each of which contained healthy control colonic biopsies along with active UC biopsies as described in Table 1. We refer to the data sets as Denson [16], Olsen [2], and Planell [3] to reflect the name of the submitter to GEO.

**Table 1:** Description of microarray data sets analyzed in this study.

Data set	GEO accession	Description	Reference
Denson	GSE10616	A pediatric patient population at Cincinnati Children’s Medical Center containing 58 arrays with 16 healthy controls, 18 ileo-colonic CD, 14 colon-only CD, and 10 UC.	[16]
Olsen	GSE9452	An adult population at University of Copenhagen containing 26 arrays with 5 healthy controls, 13 UC samples without signs of macroscopic inflammation and 8 UC samples with evidence of macroscopic inflammation.	[2]
Planell	GSE38713	An adult population in Barcelona, Spain (IDIBAPS) containing 43 arrays with 13 healthy controls, 8 inactive UC,7 noninvolved active UC, and 15 involved active UC (macroscopic inflammation).	[3]

In order to obtain the maximum statistical power for our study we aimed to pool all three datasets into one study given that they were assayed on the same microarray platform, the Affymetrix U133 Plus 2. We pooled all arrays into a single dataset of CEL-format files and performed PLIER [17], [18] probe-level analysis to quantify transcript abundance in the Affymetrix expression console. The result of that analysis is shown in Figure S1 in File S1 as the Spearman rank correlation matrix. The matrix illustrates that the arrays are highly stratified according to their origin. For instance, the Denson control arrays correlate very strongly with Denson UC samples but only weakly with Planell control arrays. We determined that site-specific differences in sample production and processing of the Affymetrix arrays made it statistically unsound to combine all arrays into a single study, leading us to opt instead to analyze each of the three data sets independently and pool the results of the final pathway analysis.

### Principal Components Analysis

In order to support the use of these datasets in elaborating the pathways that distinguish inflamed UC tissue from healthy tissue, we aimed to establish that each dataset produced consistent and well-separated patterns of gene expression.

**Figure 1:**
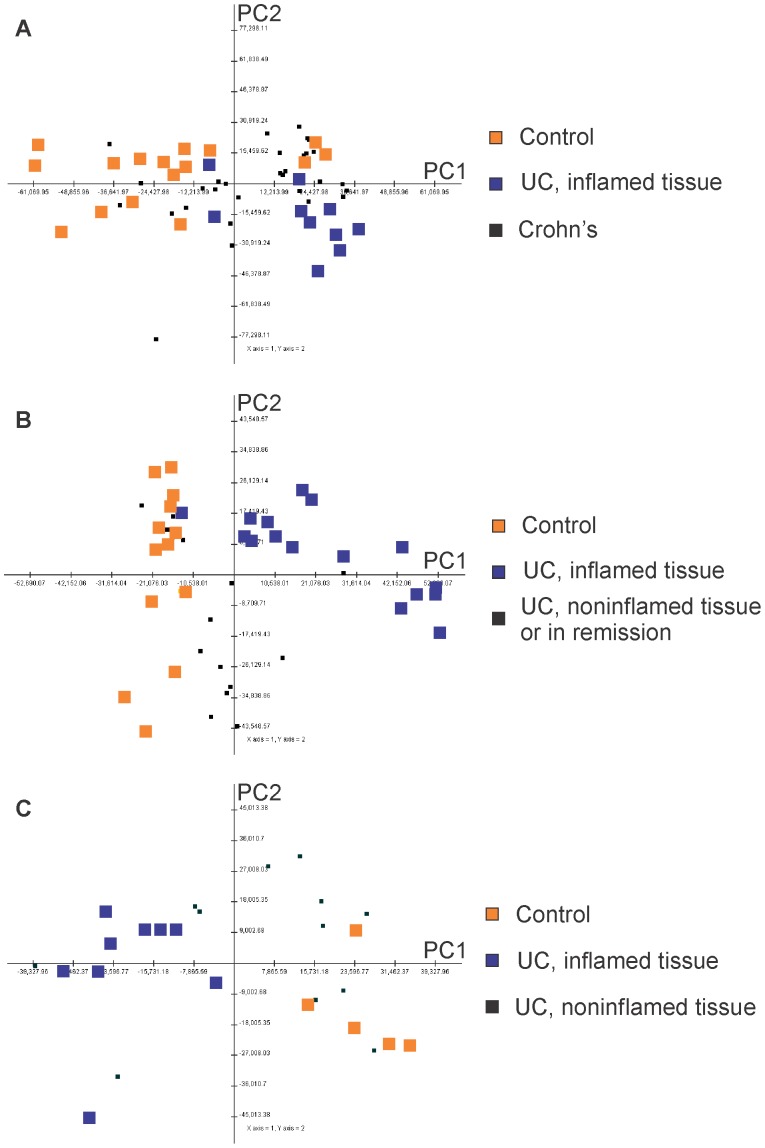
Principal components analysis of the ulcerative colitis biopsies shows that healthy control mucosa and UC inflamed mucosa exhibit distinct patterns of gene expression. The first two principal component axes are graphed, with PC1 on the horizontal axis and PC2 on the vertical axis. Healthy control biopsies are indicated by orange squares and macroscopically-inflamed UC biopsies by blue squares. All probesets on the array (54675) were included in the analysis. (A) Denson data set, black square represent Crohn’s biopsies. (B) Planell data set, black squares represent inactive UC or uninvolved UC biopsies. (C) Olsen data set, black squares represent noninvolved UC biopsies.

To create a visualization of the three datasets, we performed principal components analysis (PCA) using all probesets present on the arrays. As shown in Figure 1, the healthy control and active UC biopsies were clearly separated from one another by principal component axis 1, i.e., the axis that captures the greatest amount of variance in the data matrix. The amount of variance represented by PC axis 1 was 21% for Denson, 18% for Olsen, and 17% for Planell. This result suggests that a gene set enrichment analysis using healthy controls vs. active UC as a comparison would be valid in generating gene set lists characteristic of UC. We found that CD biopsies or biopsies of noninvolved tissue from UC patients were scattered throughout the first two principal component axes, suggesting that these specimens may or may not have inflammatory gene expression characteristics regardless of their macroscopic appearance. However, this does not rule out the possibility that more specific phenotypic parameters, not available for these GEO samples, may be account for variance in the data for CD or noninflamed UC.

### Gene Set Enrichment Analysis of Expression Data Sets

We performed an empirical Bayes testing procedure using the limma package in R to pre-rank approximately 20,000 genes by modified *t*-statistics for testing differential expression from highest (positive for upregulated) to lowest (negative for downregulated) [19]. These rank lists were used to conduct GSEA [20] contrasting UC with healthy controls, produced an abundance of upregulated gene sets in the UC biopsies and relatively fewer downregulated gene sets. We utilized the curated pathways from Biocarta, Kyoto Encyclopedia of Genes and Genomes (KEGG), and Reactome as gene sets. An example of a GSEA result for a single pathway, chemokines and their receptors, is shown in Figure S3 in File S1, showing that chemokines and their receptors are located near the top of the ranked gene list. The rank lists are provided in Tables S1 (Denson), S2 (Olsen), and S3 (Planell).

**Figure 2:**
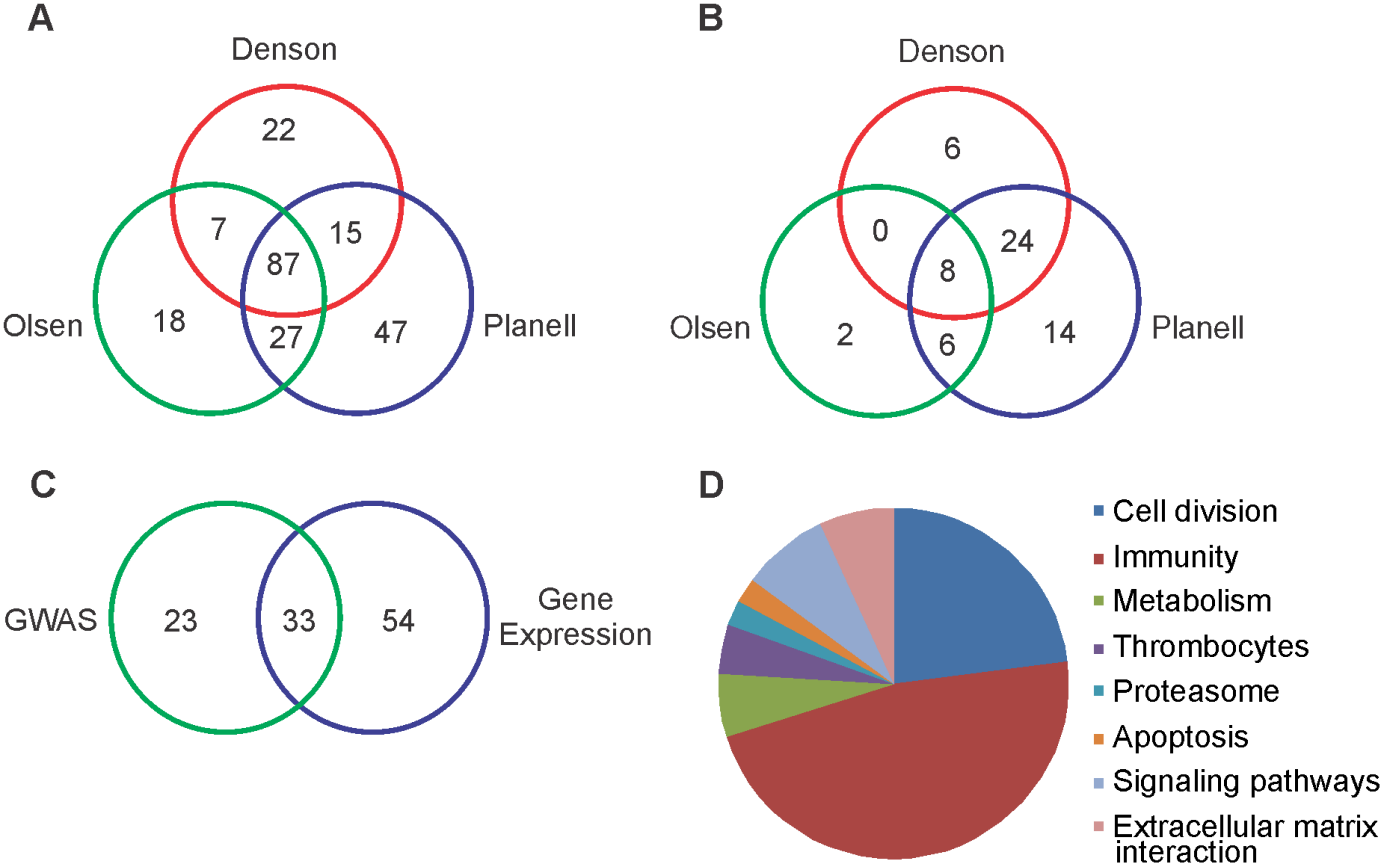
Gene set enrichment analysis (GSEA) demonstrates high concordance between three expression data sets as well as genome-wide association study susceptibility loci. (A) Venn diagram of the intersection of gene sets upregulated in the inflamed UC samples. Eighty-seven gene sets were upregulated in all three studies. (B) Venn diagram of gene sets identified as downregulated in the three studies. (C) Overlap in gene sets identified as common to the three gene expression datasets (87 gene sets) with 56 gene sets identified as having altered expression in a genome wide association study of ulcerative colitis. (D) Pie chart showing functional categories of the 87 gene sets upregulated in all three biopsy datasets.

In order to elucidate a set of common pathways, we looked for the intersection of the upregulated and downregulated pathways of all three data sets and found that 87 pathways were upregulated in active UC biopsies (Figure 2A) while 8 were downregulated (Figure 2B). The list of 87 pathways is displayed in Table 2 using the normalized enrichment score (NES) and FDR Q-values from the Denson dataset. The gene sets in this table are classified according to functional category and whether the gene set was enriched in GWAS (described below). BioCarta identified many immune-related pathways such as inflammation, natural killer T cells, cytokines, IL12, and the complement pathway. Likewise, KEGG contains many immune-related gene sets that overlap with BioCarta, but also corroborates the involvement of less-expected gene sets, such as the proteasome, apoptosis, and extracellular matrix interaction. The Reactome gene sets largely agreed with the immune-related functions, signaling pathways, and extracellular matrix interactions. However, the Reactome gene sets alone showed that in all three datasets there was an upregulation of genes involved in cell proliferation and the control of mitosis, including G1-to-S transition, synthesis of DNA, SCF/skp2 mediated degradation of p27/p21, and p53 regulation, among others.

**Table 2:** Upregulated pathways by GSEA from active UC lesions common to three gene expression data sets.

Source and Pathway	NES	Q-value	In GWAS	Category
**BioCarta**				
Cytokines and inflammatory response	2.302	0	Y	Immune
Selective expression of chemokine receptors during T-cell polarization	2.191	0	Y	Immune
Proteasome complex	2.144	0		Proteasome
Cytokine network	2.119	0.001	Y	Immune
NO-dependent IL12 pathway in NK cells	2.001	0.002	Y	Immune
Cells and molecules involved in local acute inflammatory response	1.931	0.004		Immune
Th1/Th2 differentiation	1.896	0.006	Y	Immune
Dendritic cells in regulating Th1 and Th2 development	1.818	0.012	Y	Immune
Co-Stimulatory signal during T-cell activation (CTLA4)	1.678	0.037	Y	Immune
Complement pathway	1.678	0.037	Y	Immune
Caspase cascade in apoptosis	1.675	0.037		Apopt
IL12 and STAT4 dependent signaling pathway in Th1 development	1.657	0.042		Immune
**Kyoto Encyclopedia or Genes and Genomes**				
Proteasome	2.602	0		Proteasome
Cytokine/cytokine receptor interaction	2.578	0	Y	Immune
Extracellular matrix receptor interaction	2.484	0		Mitosis
Leishmania infection	2.471	0	Y	Immune
Graft versus host disease	2.371	0	Y	Immune
Allograft rejection	2.333	0	Y	Immune
Complement and coagulation cascades	2.284	0		Immune
Protein export	2.257	0		Metab
Glycosaminoglycan biosynthesis of chondroitin sulfate	2.255	0		ECM
Type I diabetes mellitus	2.228	0	Y	Immune
Antigen processing and presentation	2.214	0	Y	Immune
Intestinal immune network for IgA production	2.182	0	Y	Immune
Asthma	2.117	0	Y	Immune
Autoimmune thyroid disease	2.106	0.001	Y	Immune
Hematopoietic cell lineage	2.084	0.001	Y	Immune
Systemic lupus erythematosus	2.054	0.001	Y	Immune
Chemokine signaling pathway	2.048	0.001	Y	Immune
Toll like receptor signaling pathway	2.031	0.001		Immune
Cell adhesion molecules (CAMs)	2.018	0.002	Y	ECM
NOD-like receptor signaling pathway	1.956	0.003		Immune
JAK STAT signaling pathway	1.911	0.005	Y	Signal
Viral myocarditis	1.761	0.021	Y	Immune
Glycosphingolipid biosynthesis, ganglio series	1.758	0.021		Metab
Focal adhesion	1.75	0.023		ECM
Pathogenic Escherichia coli infection	1.741	0.024		Immune
Natural killer cell mediated cytotoxicity	1.739	0.024	Y	Immune
**Reactome**				
Chemokine receptors bind chemokines	2.782	0		Immune
G1/S transition	2.616	0		Mitosis
Synthesis of DNA	2.6	0		Mitosis
S phase	2.599	0		Mitosis
CDT1 association with the CDC6 ORC origin complex	2.582	0		Mitosis
ORC1 removal from chromatin	2.548	0		Mitosis
DNA replication pre-initiation	2.535	0		Mitosis
Mitotic M/G1 phases	2.535	0		Mitosis
Cell cycle checkpoints	2.518	0		Mitosis
M/G1 transition	2.507	0		Mitosis
p53 independent DNA damage response	2.505	0		Mitosis
Stabilization of p53	2.504	0		Mitosis
Regulation of ornithine decarboxylase	2.5	0		Metab
Cdc20 phospho-APC mediated degradation of cyclin A	2.487	0		Mitosis
VIF mediated degradation of APOBEC3G	2.473	0		Immune
Cyclin E associated events during G1/S transition	2.45	0		Mitosis
Unfolded protein response	2.446	0		Signal
Regulation of APC activators between G1/S and early anaphase	2.435	0		Mitosis
SCF Skp2 mediated degradation of p27 p21	2.399	0		Mitosis
Autodegradation of CDH1 by CDH1 anaphase promoting complex	2.378	0		Mitosis
Signaling in immune system	2.342	0	Y	Immune
Cell cycle mitotic	2.33	0		Mitosis
SCF beta-TRCP mediated degradation of EMI1	2.313	0		Mitosis
Immunoregulatory interactions between a lymphoid and a non lymphoid cell	2.24	0	Y	Immune
Integrin cell surface interactions	2.216	0		ECM
Platelet degranulation	2.2	0		Platelet
Peptide ligand binding receptors	2.157	0		Signal
Signaling by Wnt	2.152	0		Signal
Hemostasis	2.109	0.001		Platelet
Initial triggering of complement	2.102	0.001	Y	Immune
PD1 signaling	2.099	0.001	Y	Immune
Costimulation by the CD28 family	2.059	0.001	Y	Immune
Apoptosis	2.058	0.001		Apopt
Cell surface interactions at the vascular wall	2.05	0.001		Immune
Mitotic prometaphase	2.029	0.001		Mitosis
Host interactions of HIV factors	2.027	0.001		Immune
HIV infection	2.005	0.002		Immune
G alpha i signaling events	1.925	0.004		Signal
Class A1 rhodopsin like receptors	1.922	0.005		Signal
Formation of platelet plug	1.91	0.005		Platelet
Translocation of ZAP70 to immunological synapse	1.895	0.006	Y	Immune
Metabolism of amino acids	1.891	0.006		Metab
Complement cascade	1.868	0.008	Y	Immune
Platelet activation	1.866	0.008		Platelet
Cell-extracellular matrix interactions	1.851	0.009		ECM
Generation of second messenger molecules	1.795	0.015	Y	Signal
Innate immunity signaling	1.744	0.024		Immune
Metabolism of nucleotides	1.718	0.028		Metab
Phosphorylation of CD3 and TCR zeta chains	1.676	0.037	Y	Immune

The second column gives the normalized enrichment score (NES) from GSEA analysis, the third column the FDR Q-value calculated by permutation, and the fourth column the functional category of the pathway.

Another pathway highlighted by the Reactome database is the function of platelets, which is evidenced by three gene sets being significantly upregulated, including hemostasis, formation of platelet plug, and platelet activation.

Activation of T cells by signaling through the T cell antigen receptor was represented among the significant pathways, including translocation of ZAP70 to the immunological synapse, costimulation by CD28, T cell co-stimulatory signaling, and phosphorylation of CD3 and TCR ζ chains. Cytokines, chemokines, and their receptor signaling pathways appear in 7 of the 87 pathways, indicating that diverse members of these families are enriched in the UC inflamed tissue.

Several signaling pathways were enriched among the concordant upregulated gene sets, such as JAK-STAT signaling, NOD-like receptor signaling, unfolded protein response, Wnt signaling, Gα_i_ signaling, and generation of second messenger molecules.

With regard to the downregulated pathways in mucosal gene expression, both KEGG and Reactome are in agreement that the TCA (Krebs) cycle and oxidative phosphorylation in the mitochondria are reduced in the active UC biopsies (Table 3). Two less-expected pathways, Parkinson’s disease, and regulation of insulin secretion, also appeared among the downregulated gene sets (Figure S2 in File S1).

**Table 3:** Downregulated pathways by GSEA from active UC lesions common to three gene expression data sets.

Pathway	NES	Q-value
**Kyoto Encyclopedia or Genes and Genomes**		
Citrate cycle/TCA cycle	−2.130	0.005
Parkinson’s disease	−2.083	0.006
Oxidative phosphorylation	−2.005	0.010
**Reactome**		
Electron transport chain	−2.662	0.000
Glucose regulation of insulin secretion	−2.167	0.005
Peroxisomal lipid metabolism	−2.149	0.005
Integration of energy metabolism	−1.893	0.017
Regulation of insulin secretion	−1.783	0.033

NES: normalized enrichment score from GSEA; FDR Q-value calculated from permutation.

The pie chart in Figure 2D emphasizes the role of mitotic control and immune-related pathways in differentiating gene expression profiles between healthy mucosa and UC inflammation based on the 87 upregulated concordant pathways.

Of the 1452 pathways in our curated gene sets, there are 161 immune- and inflammation-related gene sets. It is interesting to examine the result from the use of solely immune and inflammation related gene sets. We performed this GSEA analysis using only these immune gene sets and found that there were 60 pathways significant, compared with the 43 immune pathways significant when considering all 1452 pathways at the same FDR level. Thus we experience a modest loss of power due to the correction for multiple testing, however, our analysis is more comprehensive by including all pathways available.

### Characterization of the Pathway Database

We further sought to characterize our pathway database by establishing that there are 3,216 genes in common to more than 3 gene sets and 1,700 genes unique to only one gene set (Table S5). We also calculated overlap coefficients for all the pathways in our analysis and display them as heatmaps in Figure S4 in File S1. The overlap is generally small with 85% of the overlap coefficients less than 0.01.

Due to the current limited knowledge of functional pathways, pathway analysis may not be an exhaustive approach and may miss genes of unknown function but with good discriminative power. The Affymetrix U133 Plus 2 array covers 19,944 genes but the pathway gene set lists cover only 6,804 genes. We found that the percentage of differentially expressed genes are higher for the 6,804 annotated genes than for the non-annotated genes (54% vs. 42% for the Denson data set at FDR = 5%). Nevertheless, there is a clear limitation to the exhaustiveness of the analysis caused by the fact that many differentially expressed genes are not located within the pathways gene set lists.

### Genome-Wide Association Study Results

We sought greater understanding of our transcriptome findings by comparing them with results from a pathway enrichment analysis of several large GWAS SNP array datasets, described by Jostins, et al [5]. We utilized an approach to pathway discrimination based on a simple hypergeometric test that is described in Materials and Methods. The results of the gene set enrichment analysis based on GWAS loci are shown in Table 4. We set an FDR Q-value of less than 5% as a cut-off to call a gene set significantly enriched among the GWAS loci. The gene sets identified cover virtually every facet of the immune response, and most are represented among the 87 gene-expression delineated pathways (see Figure 2C). Of the 56 GWAS significant pathways, 33 are represented among the 87 gene-expression delineated pathways (hypergeometric *P* = 1.49×10^–19^ for this overlap). The column “In GWAS” of Table 2 shows which of the 87 concordant gene expression-delineated gene sets were also enriched in the GWAS pathway analysis. We also explored the overlap between gene expression and GWAS at the individual gene level in Table S4, which shows individual genes significant in gene expression, genes significant in GWAS, and the overlap between these two sets.

**Table 4:** Curated pathways found to be statistically significant by hypergeometric test in a GWAS of ulcerative colitis (FDR<5%).

**Biocarta**
**Lymphocyte activation and differentiation**
Th1 and Th2 differentiation
Antigen-dependent B cell activation
B lymphocyte cell surface molecules
The co-stimulatory signal during T-cell activation (CTLA4)
Dendritic cells in regulating TH1 and TH2 development
Activation of CSK by cAMP-dependent protein kinase inhibits signaling through the T cell receptor
Bioactive peptide induced signaling pathway
**Cytokines**
Cytokines and regulation of hematopoiesis
Cytokines and the inflammatory response
Selective expression of chemokine receptors during T-cell polarization (natural killer T cell)
IL22 soluble receptor signaling pathway
IL-10 anti-inflammatory signaling
NO-dependent IL 12 pathway in NK cells
**Complement**
Classical complement pathway
Complement pathway
Lectin-induced complement pathway
**Kyoto Encylopedia of Genes and Genomes**
**Immune-mediated diseases**
Leishmania infection
Type I diabetes mellitus
Systemic lupus erythematosus
Viral myocarditis
Asthma
Primary immunodeficiency
Allograft rejection
Graft versus host disease
Autoimmune thyroid disease
**Cellular processes**
Endocytosis
Cell adhesion molecules, CAMs
**Immune cell pathways**
Antigen processing and presentation
Intestinal immune network for IgA production
Cytokine-cytokine receptor interaction
Natural killer cell mediated cytotoxicity
Fc gamma receptor mediated phagocytosis
Hematopoietic cell lineage
**Signaling pathways**
Chemokine signaling pathway
JAK-STAT signaling pathway
NOTCH signaling pathway
**Reactome**
**Lymphocyte activation and differentiation**
Costimulation by the CD28 family
Downstream T cell receptor signaling
Generation of second messenger molecules
PD1 signaling
Phosphorylation of CD3 and TCR zeta chains
Signaling in immune system
T cell receptor signaling
Translocation of ZAP70 to immunological synapse
Immunoregulatory interactions between a lymphoid and a non lymphoid cell
**Transcription**
RNA polymerase I promoter clearance
RNA polymerase I promoter opening
RNA polymerase I, III and mitochondrial transcription
Notch-HLH transcription pathway
Transcription
**Telomeres**
Packaging of telomere ends
Telomere maintenance
**Complement**
Initial triggering of complement
Complement cascade

The Biocarta gene sets could be classified into four overall categories: immunity, lymphocyte activation and differentiation, cytokines, and complement (Table 4). KEGG gene sets consisted of immune-mediated diseases and cellular pathways, endocytosis and cell adhesion molecules, and signaling pathways. Reactome gene sets were classified as lymphocyte activation and differentiation, transcription by RNA polymerases I and III, telomeres, and complement. We have selected three pathways that produced a strong enrichment for visualization in our supplementary figures: cell adhesion molecules, cytokine-receptor interaction, and T cell receptor signaling, which are shown in Figures S5, S6, and S7.

The top normalized enrichment score in gene expression, as well as the minimal FDR Q value in GWAS analysis, was Biocarta’s inflammatory pathway. Notably, 8 of the 12 Biocarta pathways were concordant between gene expression GSEA analysis and GWAS-derived pathway enrichment analysis (hypergeometric *P* = 5.18×10^–8^ for this overlap). In contrast to our results from gene expression analysis, the downregulation in oxidative phosphorylation and upregulation of mitotic control pathways was not reflected in the GWAS-enriched pathways.

## Discussion

We obtained three publically-available datasets of UC biopsies and controls assayed on the same microarray platform. These datasets showed a clear delineation of inflammatory from healthy gene expression profiles by principal components analysis. CD samples, as well as UC in remission or colonic mucosa from UC patients that was macroscopically not involved in the inflammatory process, gave profiles throughout the inflammatory-healthy spectrum. Applying gene set enrichment analysis (GSEA) [20] to the gene expression data yielded upregulated and downregulated gene sets, with substantial overlap of all three independent datasets for key pathways.

We performed a GWAS in each of eight European-ancestry data sets, totaling 5584 UC cases and 11587 controls from the International IBD Genetics Consortium (www.ibdgenetics.org). We applied a hypergeometric test to the genes identified as significant in each of the cohorts and combined the resulting test statistics to generate a meta-analysis *P* value for each of the pathways making up our gene set database. We compared these GWAS-implicated pathways with the pathways identified through our gene expression analysis to find a large intersection between the two methodologies (Figure 2C). The vast majority of the statistically significant pathways concordant among the two data sources were in the category of immune system function.

In the gene expression data we observed downregulation of gene sets involved in mitochondrial function, electron transport chain, oxidative phosphorylation, and the Krebs cycle. However, this pathway signature was not reflected in SNP-based GWAS, suggesting that perturbation of the mitochondrial respiration as well as elevated levels of cell proliferation are secondary features of inflammation in the tissue and not primary etiopathologic drivers that initiate the immune response.

It is commonly believed that the risk of autoimmune or inflammatory disease that is represented in the GWAS loci is not located in coding variation in the exome but rather in non-coding regulatory elements that flank the functionally responsible genes [21]. Whether these associations are the result of a small number of common variants at the locus or an ensemble of rare variants is open to debate [22], [23], but in either scenario, the most compelling explanation for the existence of the majority of GWAS signals is that such variants participate in transcriptional regulation independent of whether they reside in intergenic or genic regions without direct impact on coding sequences.

One may argue that the pathway enrichment seen in the gene expression dataset is merely the result of an infiltration of immune cells (lymphocytes, polymorphonuclear cells, macrophages, and dendritic cells) into the tissue which produces this gene expression signature. Moreover, microscopic levels of inflammation or histological changes in the mucosa in macroscopically normal or healed tissue may influence the profile of transcripts present in the biopsy. In the Reactome gene sets we observed an unmistakable upregulation of gene expression in mitosis control pathways suggesting that a population of cells in the macroscopically inflamed UC tissue is actively proliferating. The biopsy tissue is an admixture of various hematopoietic cell types (particularly in inflamed tissue) as well as intestinal mucosal cells from the epithelium and lamina propria. However, corroboration by GWAS signals demonstrates that the admixture of inflammatory cells is not the only factor in the gene expression pattern, but that variation in the transcriptional regulation of the immune response genes also contributes to the overrepresentation of these transcripts in the UC biopsies. UC patients are likely to be over-expressors of the inflammatory genes and this can be seen in the hybrid pattern of gene expression observed in quiescent or noninvolved UC biopsies.

An interesting observation was that certain pathways were downregulated in all gene expression datasets that were not reflected in GWAS. Two of these pathways that might be less expected are identified in this analysis: Parkinson’s disease (KEGG) and insulin secretion (Reactome). Figure S2 in File S1 shows KEGG’s curated pathway for Parkinson’s disease and illustrates that mitochondrial function, oxidative phosphorylation, proteasome function, and apoptosis are all constituents of the pathway. Hence, there may be an overlap between the processes of apoptotic cell death in the substantia nigra of the brain in Parkinson’s disease and the cell death pathway that normally occurs in the inflamed mucosa. The insulin secretion pathways are similarly involved due to the role of oxidative phosphorylation in sensing the level of ATP and producing appropriate insulin release, evidenced by the enrichment of cytochrome oxidases and NADH dehydrogenase (ubiquinone) Fe-S proteins in this gene set. The overall inference from these 8 downregulated data sets is that the inflammatory process inhibits the normal production of energy in the form of ATP and the normal processes of cell proliferation and renewal that are found in the healthy colonic mucosa. It is likely that the inflamed tissue has a reduced amount of this metabolic activity, possibly because normally energy-consuming colonic mucosa is replaced by necrotic or fibrotic tissue and infiltrated with immune cells.

In obtaining colonic biopsy samples for study, there is generally considerable variation between clinicians in which sites in the colon are sampled, as well as variation among the different research institutes in the technical production of microarray data. It is therefore a pleasing result to observe such a high degree of agreement among the three independent gene expression data sets as to which pathways are enriched in the inflammatory state. It can be concluded that UC presents a unique signature of histology and gene expression, a specific type of alteration in the mucosal immune homeostasis, which is corroborated by previous work [24]–[27].

Pathway-based analyses such as GSEA have several attractive features, namely, that they are more robust when trying to aggregate results from studies performed on different platforms, or, in this case, different study designs (gene expression vs. GWAS). The conclusion that is supported by all four data sources used in this study is that immune response genes are overwhelmingly overrepresented and overexpressed in patients with UC, particularly in the actively inflamed colonic mucosa.

The GSEA approach is a “competitive” method which tests the null hypothesis that the expression distribution of genes in a specific gene set is the same as other genes not in the pathway (or in the other pathways under testing, more precisely) [28], [29]. Therefore the gene set collection plays a critical role in our pathway-based analyses, with a profound impact in determining the null distribution and the extent of multiple testing correction. For example, when using solely the immune and inflammation related gene sets we identified fewer significant immune pathways. However, it is noted that the null hypothesis should be determined beforehand. That is to say we should not change the gene sets collection retrospectively after we observe the testing results for “improving” power.

Another issue we must consider is that pathway-based analyses will tend to favor genes which are well-studied and about which solid biological knowledge exists. Certainly there are many transcripts of unknown function which are overexpressed and there are many GWAS-implicated loci which contain no functionally obvious candidate, or more than one plausible candidate. Any database of curated gene sets is certain to be incomplete due to the current state of biological knowledge. Nevertheless, these gene set-based analyses do have value because they can prioritize and direct interest towards well-studied, therapeutically-tractable pathways that are not widely appreciated or well-integrated into our understanding of IBD pathogenesis. This being said, there are several caveats to be aware of when considering investigation of non-immune pathways. In our GWAS-based pathway enrichment study, we found several gene sets from Reactome involving transcription by RNA polymerase I and III, as well as telomere maintenance, being significantly enriched in UC (Table 4). However, inspection of the specific genes that were identified in transcription and telomeres shows that their significance was driven by the presence of many genes from the histone cluster on chromosome 6, which lies in close proximity to the HLA locus on that chromosome. HLA gives the strongest signal in GWAS of UC, therefore, nearby genes in gene rich clusters, such as the histone cluster, may contain several low *P* value SNPs in linkage disequilibrium which will give false positive signals.

Finally, our approach correlates genomic variants with transcriptome regulation. A similar approach is eQTL analysis. eQTL may be a more powerful tool to pinpoint how the genome sequence interacts with the functional genome. However, both mRNA expression data and genotype data are required for the same individuals at the same time. It first imposes a cost issue when both transcriptome and genome data have to be produced. Since different projects have different aims; most GWAS/genetics projects usually don’t profile transcriptomes. There is no transcriptomic profiling in the study by the International IBD Genetics Consortium [5] from which we obtained in part the genotype data for our current work. Conversely, a great many molecular biology projects generate mRNA data but don’t consider nor generate any genotype data, as evidenced by the majority of microarray data hosted at the NIH GEO database, including the ones we used. As of today, mRNA expression data and genotype data are accumulated in parallel with little overlap (i.e., eQTL data is still a minority in comparison with pure mRNA data or pure genotype data). Our work, as a proof of principle, shows that we may fully exploit these ample existing separated datasets to conduct secondary analysis. Such an approach, even though it may be a pure categorical overlap, has the ability to produce additional interesting findings missed in their first-round analysis at no extra data cost, as these two kinds of data are complementary to each other.

In conclusion, we propose and correlate for the first time GWAS data with independent transcriptome data at pathway-level. Our GSEA results highlight several pathways which have not be thoroughly investigated in ulcerative colitis and which may be of interest to the clinical molecular biology community, including endocytosis and extracellular matrix interaction. Analysis of gene expression in the biopsies further implicates mitotic control, the ubiquitin/proteasome system, hemostasis by platelets, and numerous signaling pathways.

## Materials and Methods

### Ethics Statement

Gene expression data was obtained from a public repository administered by the U.S. National Institutes of Health, the Gene Expression Omnibus (GEO). The repository is available at http://www.ncbi.nlm.nih.gov/geo/. Data contained within GEO is anonymized and de-identified by the submitters before it is uploaded to the repository. All studies are approved by the Institutional Review Board of the Children’s Hospital of Philadelphia.

### Data Sets and Probe-level Analysis

In the three publically-available data sets, the healthy control samples were obtained from individuals undergoing routine screening colonoscopy or from individuals with irritable bowel syndrome-like symptoms who were diagnosed as not IBD. The Denson dataset has not been subjected to a systematic analysis before and is uniquely derived from children under the age of 18. Affymetrix CEL files from the Human Genome U133 Plus 2.0 arrays were downloaded from the Gene Expression Omnibus (National Center for Biotechnology Information) using the GSE accession numbers described in Table 1. This chip targets the 3′ end of a set of over 54,000 transcripts representing all known protein-coding genes. The CEL files were converted to probeset intensity calls in the Affymetrix Expression Console build 1.3.1.187 using the command PLIER workflow.

### Principal Components Analysis and Hierarchical Clustering of Expression Data

The raw PLIER intensity calls were imported in MultiExperiment Viewer (TMEV) version 4.8.1. Rows (genes) were normalized to have mean 0 and variance 1. PCA was run to cluster samples using all probesets on the array without filtering and the first two principal component dimensions were graphed for each of the three data sets.

### Gene Set Enrichment Analysis (GSEA)

GSEA makes it possible to compare diverse data sets, even data sets from different platforms, by identifying differentially regulated pathways based on prior biological knowledge of gene function. This analysis works by ranking the genes according to a chosen metric, in our case the log-odds of differential expression, and then analyzing all the gene sets to determine whether the members of the set are overrepresented at the top of the ranked list, the bottom, or some random pattern. The result of this calculation is an enrichment score which is normalized to the size of the gene set (NES). The false discovery rate Q-value is computed by comparing the tails of the observed and null (i.e., randomly permuted) distributions of the NES [20]. We utilize an FDR cut-off of 5% for calling a gene set differentially regulated.

The Affymetrix CEL files were imported into Bioconductor in the R statistical computing environment using RMA probe-level analysis. The limma package (Linear Models for Microarrays) was used to create a pre-ranked list using the eBayes function, the empirical Bayesian testing procedure with modified *t* statistics [19]. The pre-ranked list was analyzed in GSEA software using the default parameters, namely: normalization mode meandiv; enrichment statistic weighted; collapsing mode maxprobe; minimum set size 15; maximum set size 500; and 1000 permutations. We utilized the gene set database of all curated pathways, “c2.cp.v3.0.symbols.gmt”.

Venn diagrams of the intersections of the enriched gene sets were constructed by custom code written in Python.

### Genome-Wide Association Study and GSEA

The GWAS pathway study covers seven GWAS cohorts genotyped on conventional SNP arrays and one cohort genotyped on the Immunochip, a custom Illumina Infinium chip. This custom chip is designed to perform both deep replication of suggestive associations and fine mapping of established GWAS significant loci. It provides a more comprehensive catalog of the most promising candidate variants by picking up the remaining common variants and rare variants that are missed in the first generation of GWAS. The data set was obtained from the International IBD Genetics Consortium (www.ibdgenetics.org) and included their ulcerative colitis cohorts (Cedars, CHOP, Germany, NIDDK1, NIDDK2, Norwegian, Swedish, WTCCC, and ImmunoChip).

PLINK [30] was used to conduct single SNP association analysis. Standard quality control procedures were used to remove SNPs out of Hardy-Weinberg equilibrium or with low frequency and logistic regression was used to correct for population stratification with multidimensional scaling dimensions used as covariates [5]. Gene-level association significance was determined by taking the minimal *P* value of the SNPs in a gene region (defined as 100 kb upstream and downstream of the gene boundary) adjusted by the number of SNPs (Bonferroni correction) in the gene region. We used a *P*-value cutoff of 0.05 to claim a gene to be significant, which then divided all genes into two groups, the significant group vs. the non-significant group, for each of the eight cohorts. These significant vs. non-significant gene categories were used as the basis of a hypergeometric test for evaluating pathway enrichment of significant genes in a given gene set. This pathway enrichment analysis was conducted separately for each cohort. Individual significances were then combined into a summary meta *P*-value across all participating cohorts. We used the Benjamini-Hochberg [31] procedure for pathway-level multiplicity control and claimed pathways to be significant under a false discovery rate (FDR) cutoff of 5%.

## Supporting Information

Figure S1 **Figures S1-S4.**
(DOCX)

Figure S5 **KEGG pathway “cell adhesion molecules” with genes significant in GWAS highlighted by red text.**
(PNG)

Figure S6 **KEGG pathway “cytokine-cytokine receptor interaction” with genes significant in GWAS highlighted in red text.**
(PNG)

Figure S7 **Reactome pathway “T cell receptor signaling” with genes significant in GWAS highlighted in yellow.**
(PNG)

Table S1 **Rank list of Denson biopsy data set for differential expression by empirical Bayes testing procedure.** Genes are ordered by modified *t* statistics, with positive values representing upregulation and negative values representing downregulation. These rank lists served as the input for the Gene Set Enrichment Analysis (GSEA) on microarray data. The ranks were calculated by comparing healthy controls with inflamed tissues from ulcerative colitis biopsies.
(XLSX)

Table S2 **Rank list of Olsen biopsy data set.**
(XLSX)

Table S3 **Rank list of Planell biopsy data set.**
(XLSX)

Table S4 **Gene-level overlap between gene expression and GWAS data.** Column A lists the genes which are differentially regulated in gene expression in all three data sets (Denson, Olsen, Planell). Column B lists the genes with adjusted *P* values below 0.05 in meta-analysis. Column C is the intersection between Column A and B.
(XLSX)

Table S5 **A large proportion of genes making up the curated gene sets (Biocarta, KEGG, and Reactome) appear in 3 or more gene sets.** Column A gives the list of genes appearing in 3 or more gene sets. Column B lists genes unique to only one gene set.
(XLSX)
