# Supplementary material for: Transcriptome Profiling of Human Ulcerative Colitis Mucosa Reveals Altered Expression of Pathways Enriched in Genetic Susceptibility Loci
Source: PLoS One. 2014 May 1;9(5):e96153. doi: 10.1371/journal.pone.0096153 (PMC4006814; doi:10.1371/journal.pone.0096153)
Supplement: Figure S1 — Figures S1-S4. (DOCX) [file pone.0096153.s001.docx]

# Supplementary Information

Transcriptome profiling of human ulcerative colitis mucosa shows altered expression of pathways overlapping genome-wide association susceptibility loci

Christopher J. Cardinale^1 *^, Zhi Wei^2 *^, Jin Li^1^, Junfei Zhu^2^, Mengnan Gu^2^, Robert N. Baldassano^3,4^, Struan F.A. Grant^1,4^ and Hakon Hakonarson^1,4 ‡^

^1^ Center for Applied Genomics, ^3^Division of Gastroenterology, Hepatology, and Nutrition, Children’s Hospital of Philadelphia and ^4^Department of Pediatrics, Perelman School of Medicine at the University of Pennsylvania, Philadelphia, PA 19104, USA

^2^ Department of Computer Science, New Jersey Institute of Technology, Newark, NJ 07102, USA

* These authors contributed equally

# ‡ Correspondence:

Children’s Hospital of Philadelphia Research Institute, Abramson Research Center Suite 1216, 3615 Civic Center Boulevard, Philadelphia, PA 19104.

E-mail: hakonarson@chop.edu, Telephone: 267-426-6047, Fax: 267-426-0363.

This PDF file includes:

Supplementary Figures S1–S4

Legends to Supplementary Tables S1–S7

# Figure S1. Spearman rank correlation matrix demonstrates that the three gene expression datasets performed at different institutions show a high degree of intra-institution correlation.

The matrix was generated in Affymetrix Expression Console as a quality control step and shows how the gene expression profile of each array correlates with every other array. The red end of the scale signifies a high degree of correlation while the blue end signifies low correlation. The diagonal shows that an array has a correlation of 1.0 with itself. It is notable that there is more correlation among all samples performed at a given site (for instance the Denson dataset) than there is between control samples at all sites or UC samples at all sites. This high degree of stratification by dataset requires that each dataset be analyzed separately and expression values from different sites cannot be pooled in the analysis despite their being performed on the same microarray platform.


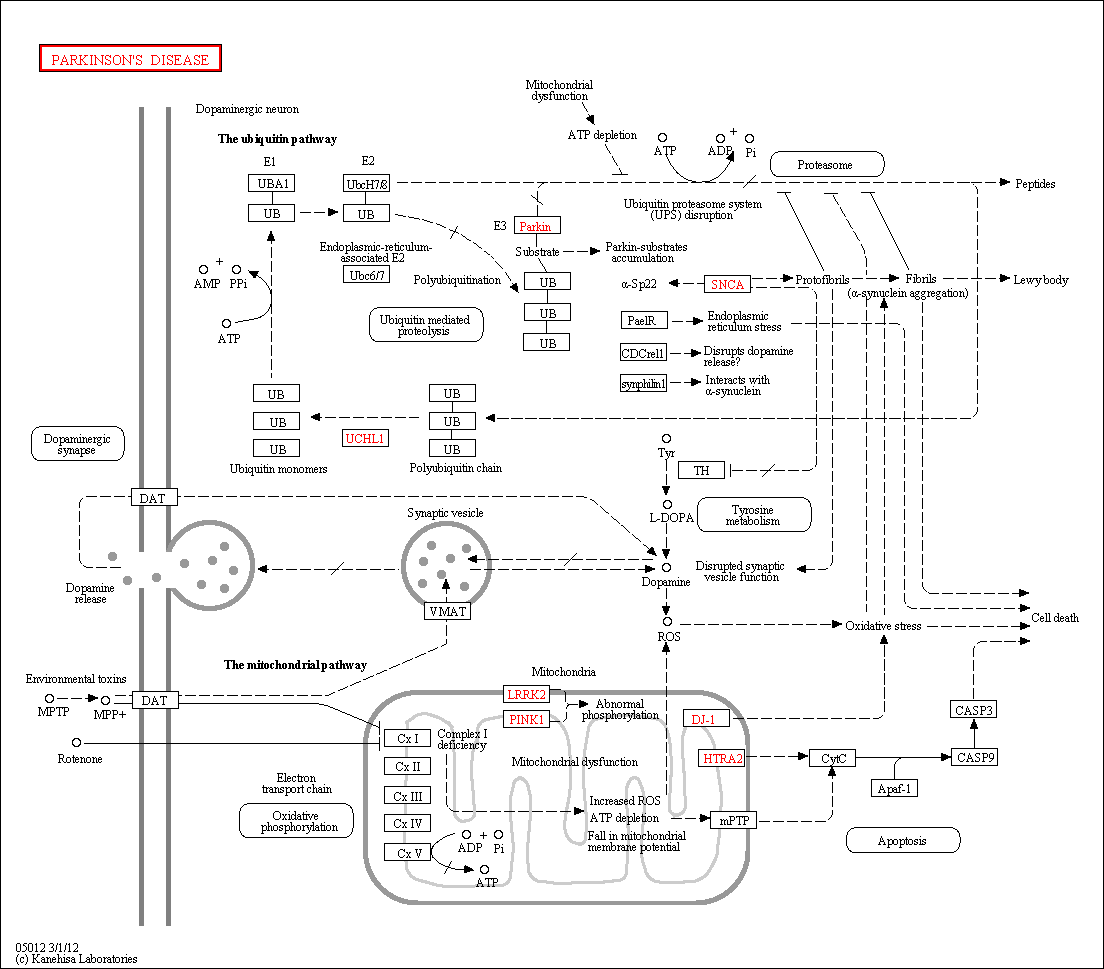


# Figure S2. Kyoto Encyclopedia of Genes and Genomes manually curated pathway for neurodegeneration in the substantia nigra due to Parkinson’s disease.

This pathway was significantly downregulated in the ulcerative colitis samples of all three datasets compared to healthy controls. The likely reason for this gene set enrichment is the presence of genes which were found in several key pathways, namely oxidative phosphorylation, electron transport chain, apoptosis, and ubiquitin/proteasome.

#
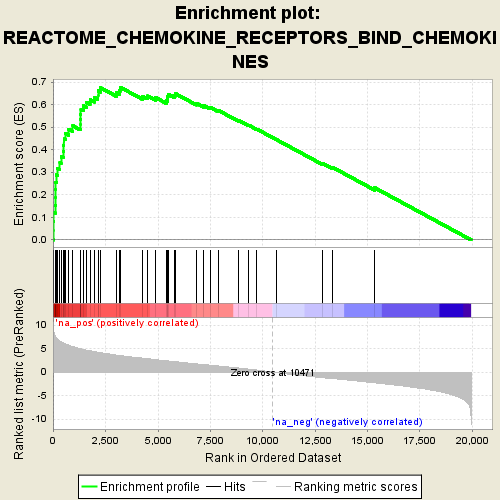


# Figure S3. Enrichment plot for Reactome “chemokine receptors bind chemokines” geneset.

Thirty-one of the 51 members of this gene set showed core enrichment in the Denson data set. The Rank in the gene list is where the gene ranks from 1 to 19944 when sorted by degree of differential expression from most upregulated to most downregulated. The rank metric score is a measure of how differentially expressed the gene is based on empirical Bayes testing procedures. The ES (enrichment score) determines whether the gene set has a disproportionate number of members at the top or bottom of the rank list.

| 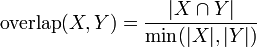 | |
| --- | --- |
| 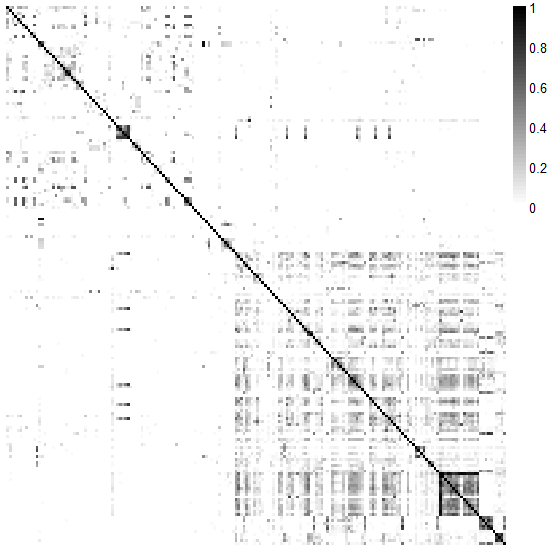  KEGG | 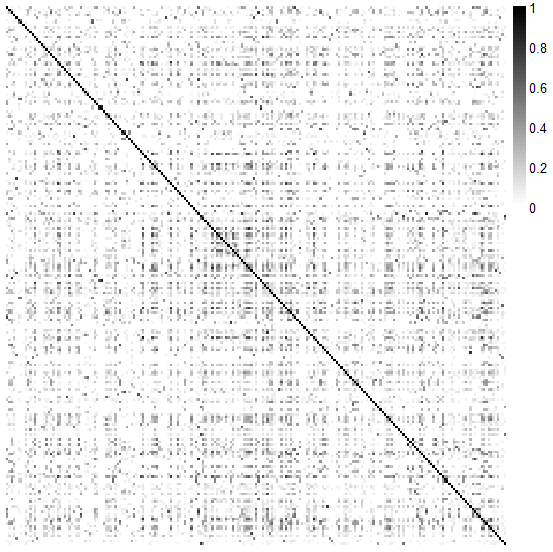  Biocarta |
| 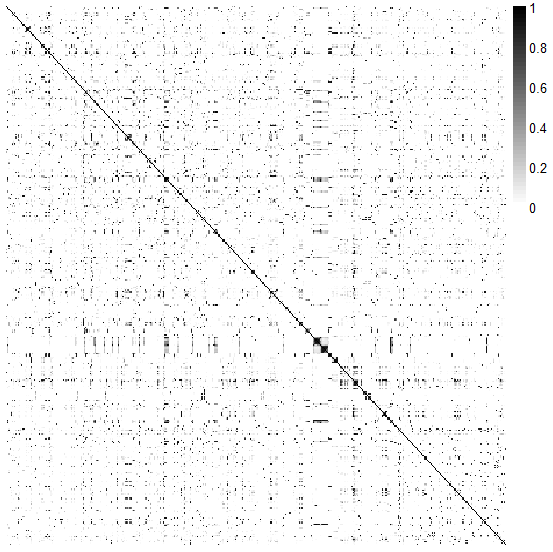  Reactome | 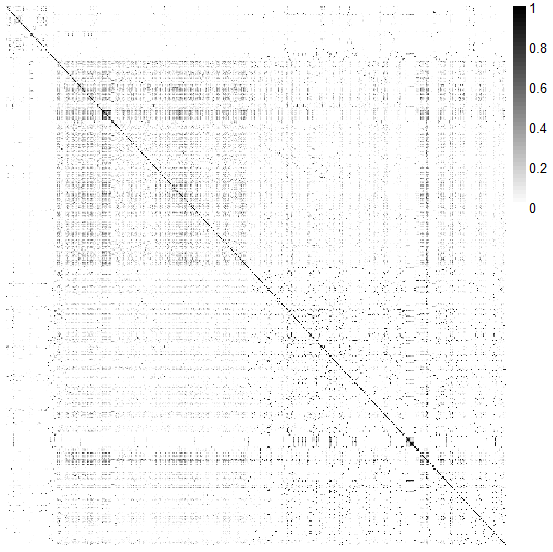  Total |

# Figure S4. Heatmaps of overlap coefficients illustrating the extent of overlap of gene sets used in this analysis.

The overlap coefficient is defined as the size of the intersection divided by the smaller of the size of the two sets. If X is a subset of Y or the converse the overlap coefficient is equal to 1. The overlap is generally small with 85% of the overlap coefficients < 0.01.
