# Supplementary figures and images for: Transcriptome Profiling of Human Ulcerative Colitis Mucosa Reveals Altered Expression of Pathways Enriched in Genetic Susceptibility Loci
Source: PLoS One. 2014 May 1;9(5):e96153. doi: 10.1371/journal.pone.0096153 (PMC4006814; doi:10.1371/journal.pone.0096153)

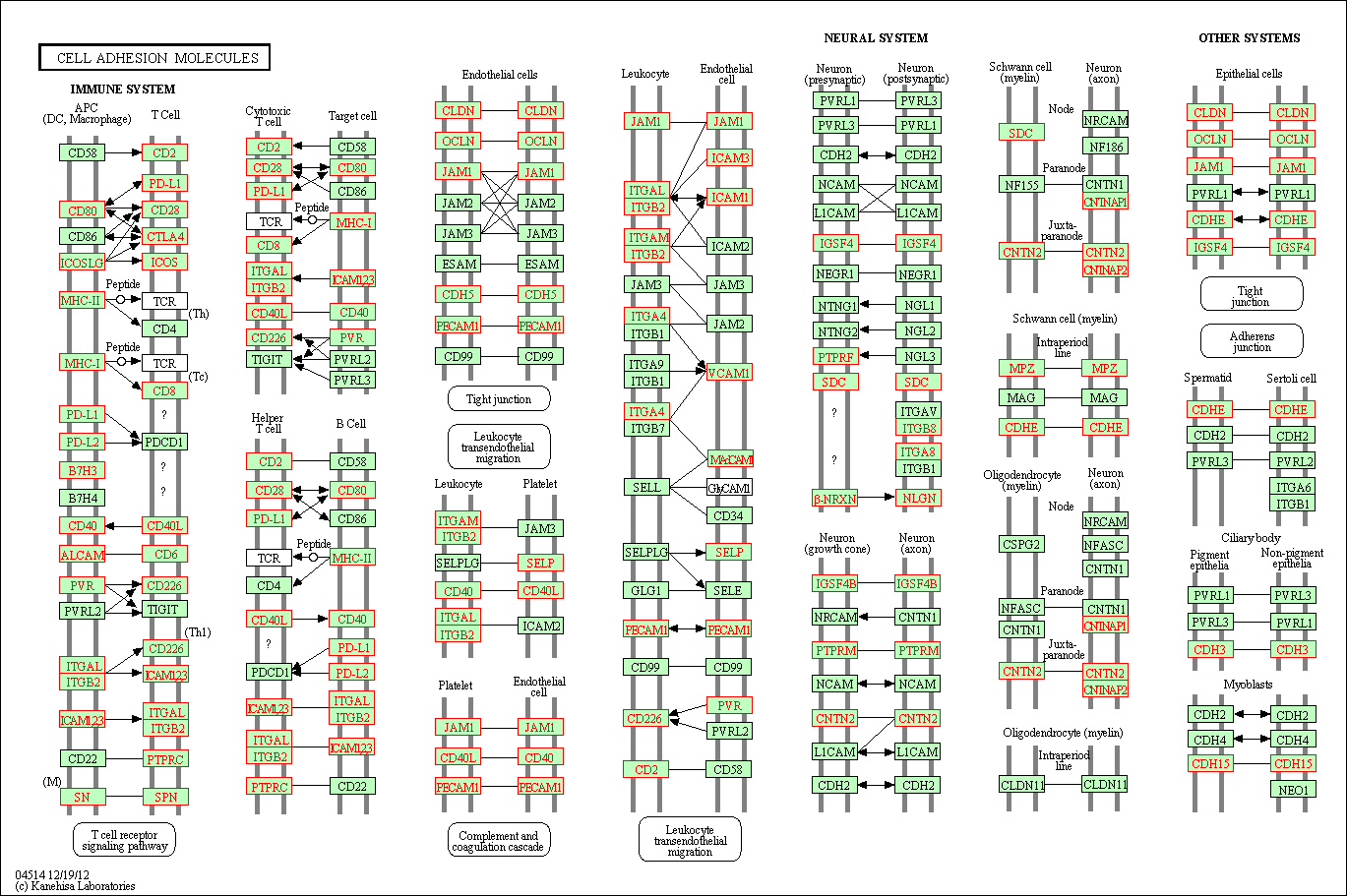

Supplement: Figure S5 — KEGG pathway “cell adhesion molecules” with genes significant in GWAS highlighted by red text. (PNG) [file pone.0096153.s002.png]

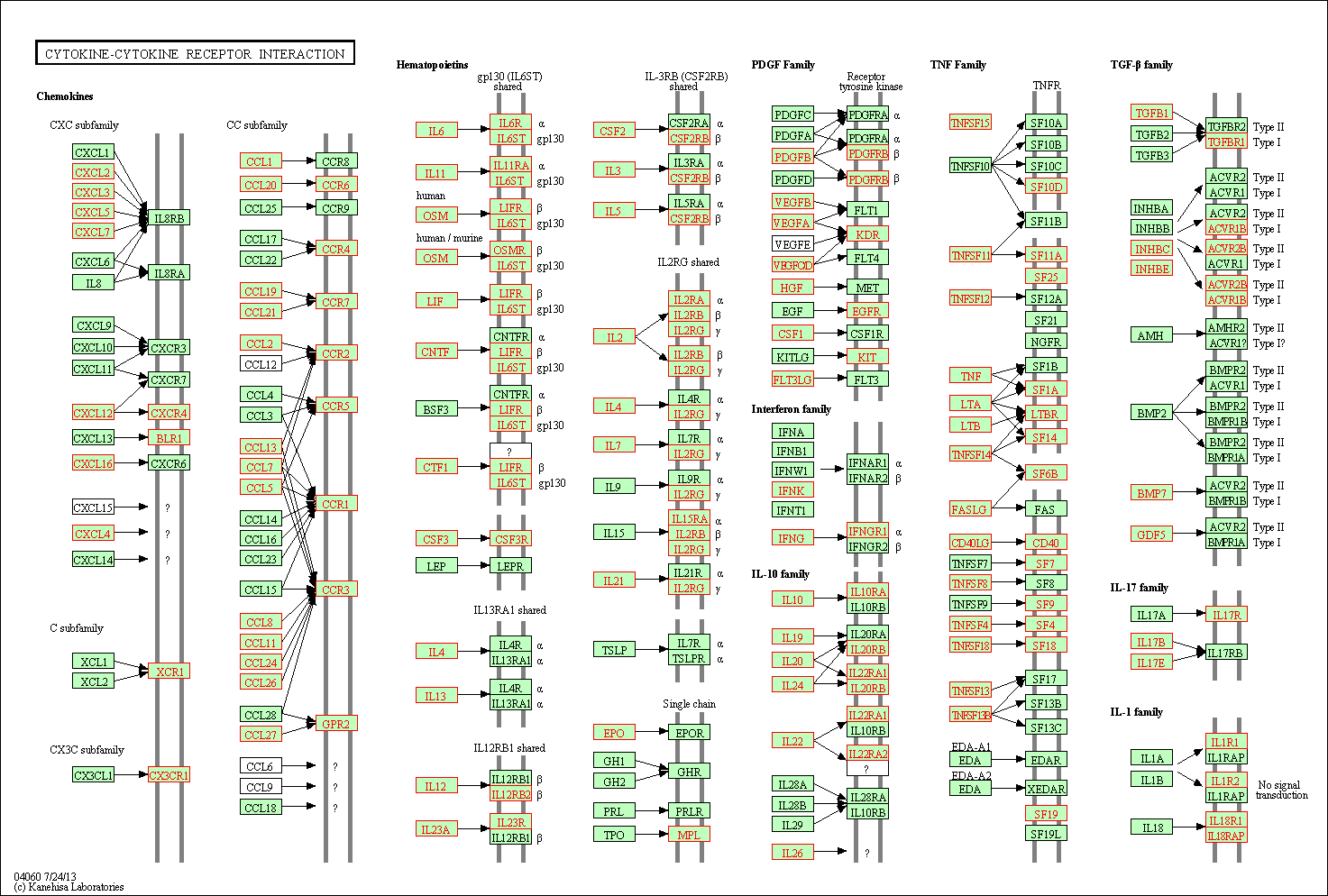

Supplement: Figure S6 — KEGG pathway “cytokine-cytokine receptor interaction” with genes significant in GWAS highlighted in red text. (PNG) [file pone.0096153.s003.png]

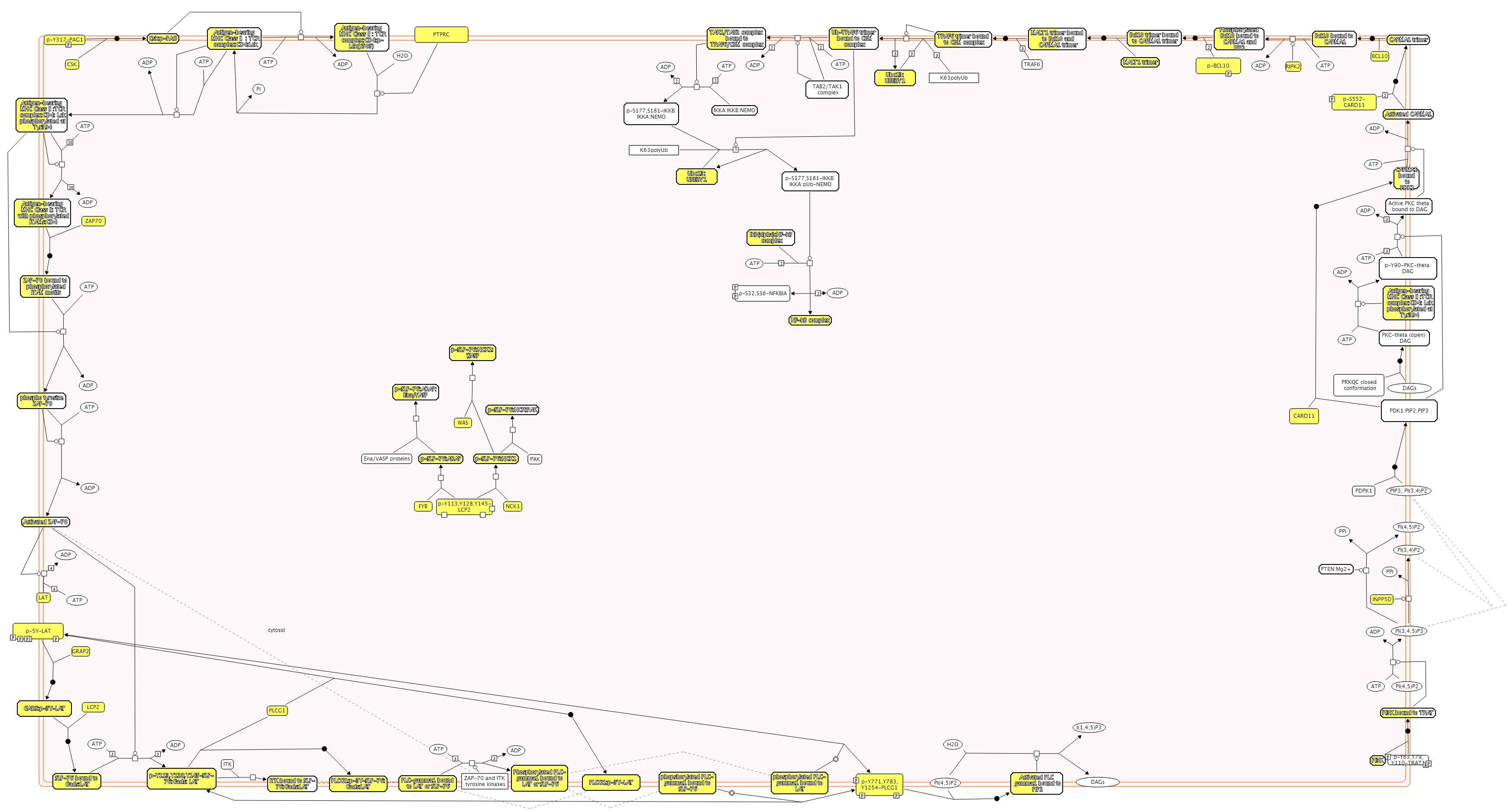

Supplement: Figure S7 — Reactome pathway “T cell receptor signaling” with genes significant in GWAS highlighted in yellow. (PNG) [file pone.0096153.s004.png]
